# Supplementary material for: When performance is not enough—A multidisciplinary view on clinical decision support
Source: PLoS One. 2023 Apr 24;18(4):e0282619. doi: 10.1371/journal.pone.0282619 (PMC10124862; doi:10.1371/journal.pone.0282619)
Supplement: Attachment — Submitted filename: PLOS_One_RebuttalLetter.pdf [file pone.0282619.s001.pdf]

Dear reviewers,

Thank you for your valuable feedback! In the following, you can find your comments and suggestions and directly below our answers.

All the best,
Roland Roller

Reviewer #1:

-Related work needs to be updated more followed by adding references.

Answer: *We updated the related work.*

-It is recommended to add a table of past work on clinical decision support.

Answer: *Literature on clinical decision support has exploded over the last years with around 10000 articles in PubMed per year in 2021 and 2022 using the search "clinical decision support" and still 1000 per year when searching "artificial intelligence clinical decision support". The challenges differ significantly depending on the clinical context, in which clinical decision support is implemented. For example in radiology, pathology, and dermatology, image analysis is used mostly, while in patient-centered approaches, chatbots and symptom checkers have been studied. In clinical settings such as ours, ML-based models are used for prediction often. Providing a general overview of clinical decision support is way beyond the scope of this article. Instead, we will focus on existing decision support in the field of kidney transplantation.*

We added a table with different works on AI-based decision support in kidney transplantation.

-Improve the quality of figure1.

Answer: *Done*

-Illustrate your clinical decision support model as figure 2.

Answer: *We included a figure to illustrate the model (currently Figure 3).*

-Brief on field of nephrology.

Answer: *We more specifically discussed related work in the area of kidney transplantation, since it is more relevant to our work than other areas in nephrology.*

-Justify why authors chose this area of Nephrology while many areas also require urgent clinical decision support.

Answer: *The main contribution of our work were the lessons learned, which are - we hope - domain-independent. However, we discussed the lessons learned, given our example in nephrology. Without a use case, some readers might wonder why or how we came up with them. But the use case itself? Well, this is the medical domain of various co-authors. Without them, it would not have been possible to access this data in the first place, and without their expertise, it would have been more difficult to set up such a system.*

-The statement in the paper required refrences to quote "we present here was developed in a project by some of the authors of this paper, namely the computer scientists and the clinical experts"

Answer: *There is little related work that is comparable and even the slightly related solutions are so different that a simple comparison would lead to confusion if anything.*

-Contrast your work with the existing or recent works furnished and prove your model to be most significant.

Answer: *We updated the related work section and included a table to prove that our model is most significant.*

-Showcase your Risk prediction model as data flow diagram.

Answer: *We included a figure to illustrate the risk prediction model as a data flow diagram (currently Figure 1).*

-Mention the ability of your model to target other areas in the scope for future work.

Answer: *We mention this in the text, and we did. Please see towards the end of the Methods section (reference 32).*

Reviewer #2:

1. Abstract is too short

Answer: *We extended the abstract.*

2. Introduction need to be improved more add some lines about the motivation about the field of study

Answer: *We added a paragraph about the motivation from a clinical perspective, where balancing the risk of infection and rejection is important and could be assisted by AI-based risk prediction models.*

3. add major contribution points at the end of the introduction

Answer: *We added major contribution points at the end of the introduction.*

4. Add literature study section after introduction and cite at least 10 papers also add one table which need to have limitation of the existing field of study.

Answer: *We updated the related work accordingly.*

5. A case study need to be explained more

Answer: *The new manuscript includes more details about the case study.*

6. Explain more figure 1

Answer: *We provided a longer explanation of (former) Figure 1.*

7. Table 1 need to be explained more in detail

Answer: *We added some more information to clarify Table 1.*

8. Add methodology section also work on some more machine learning classifiers

Answer: *We restructured the manuscript. We introduced a "data and methods" and a "result" section. However, we would like to note that originally our main intention was to present lessons learned along with our risk prediction system. The risk prediction system and machine learning were not the main focus of the work. Now we changed the document according to your suggestions. The current manuscript still emphasizes the lessons learned, but the risk prediction system itself became also more prominent.*

9. Related work is too short

Answer: *We extended the related work section.*

10. Add future directions separate section

Answer: *We included some future directions in the conclusion (as reviewer 3 suggested)*

11. Add some proper experimental results

Answer: *We restructured the document and extended the experimental results. (And similarly, as the reply for number 8, the core contributions of our work are the lessons learned)*

12. Overall paper English is too much poor try to improve the language

Answer: *We put much effort into improving the language aspects.*

Reviewer #3:

In this paper, authors presented a multidisciplinary view on machine learning in medical decision support systems. However, there are some limitations that must be addressed as follows.

1. This work is not presented correctly. The standard format of the research article should be used: abstract, introduction, related work, methodology, results, and conclusion.

Answer: *We changed the structure of the article accordingly. However, we would like to mention that originally the main focus of our article was the lessons learned in the context of decision support - which has been presented given the example of infection detection. The new manuscript makes the infection use case much more prominent. However, the lessons learned are still a substantial part of our work, therefore the discussion section is quite long.*

2. The abstract is very short and not attractive. The novelty should be clearly discussed. In addition, In the last lines of abstract, the authors should discuss results and highlight in what %age and in what parameters the proposed methodology is better as compared to existing techniques and what is the overall analysis of proposed methodology.

Answer: *We improved the abstract of this work. But again, our work is not about presenting a fancy and superior machine learning model. Instead, our model intended to present all the challenges and learning we made during the development of our system so that others can learn from it. The score of our system is not bad (and we are better than physicians) - however, this is not the focus of the work. Therefore we believe that a discussion about technical details like parameters of the methodology or “is better as compared to existing techniques” would not match the main target of our work. Still, we have tried to make the abstract more attractive by clarifying exactly this and adding some more discussion.*

3. In Introduction section, it is difficult to understand the novelty of the presented research work. This section should be modified carefully. In addition, the main contribution should be presented in the form of bullets.

Answer: *We update the introduction accordingly.*

4. More related work should be included about clinical decision support system (‘An intelligent healthcare monitoring framework using wearable sensors and social networking data’, ‘Automatic detection of Alzheimer’s disease progression: An efficient information fusion approach with heterogeneous ensemble classifiers’, ‘ANAF-IoMT: A Novel Architectural Framework for IoMT-Enabled Smart Healthcare System by Enhancing Security Based on RECC-VC’,’ Fine-Tuned DenseNet-169 for Breast Cancer Metastasis Prediction Using Fast AI and 1-Cycle Policy’). In addition, In the last lines of Literature review, highlight what overall technical gaps are observed in existing works that led to the design of proposed methodology.

Answer: *We extended the related work section. However, literature on clinical decision support has exploded over the last years with around 10000 articles in PubMed per year in 2021 and 2022 using the search "clinical decision support" and still 1000 per year when searching "artificial intelligence clinical decision support". The challenges differ significantly depending on the clinical context, in which clinical decision support is implemented. For example in radiology, pathology, and dermatology, image analysis is used mostly, while in*

patient-centered approaches, chatbots and symptom checkers have been studied. In clinical settings such as ours, ML-based models are used for prediction often. Providing a general overview of clinical decision support is way beyond the scope of this article. Instead, we will focus on existing decision support in the field of kidney transplantation.

We added a table with different works on AI-based decision support in kidney transplantation.

5. Captions of the Figures and tables not self-explanatory. These captions should be self-explanatory, and clearly explaining the figure and table. Extend the description of the mentioned figures and tables to make them self-explanatory.

Answer: *The captions have been extended.*

6. Figure 1 is blurred, its quality should be improved.

Answer: *Quality has been improved*

7. Data analysis section should be extended by including more details.

Answer: *Our new manuscript now includes more details.*

8. The conclusion section should be revised. In addition, the future work should be included.

Answer: *The conclusion has been revised and extended by future work.*
